# Expression of Phosphocitrate-Targeted Genes in Osteoarthritis Menisci

**DOI:** 10.1155/2014/210469

**Published:** 2014-11-23

**Authors:** Yubo Sun, David R. Mauerhan, Nury M. Steuerwald, Jane Ingram, Jeffrey S. Kneisl, Edward N. Hanley

**Affiliations:** ^1^Department of Orthopedic Surgery, Carolinas Medical Center, P.O. Box 32861, Charlotte, NC 28232, USA; ^2^Molecular Biology Core, Cannon Research, Carolinas Medical Center, P.O. Box 32861, Charlotte, NC 28232, USA

## Abstract

Phosphocitrate (PC) inhibited calcium crystal-associated osteoarthritis (OA) in Hartley guinea pigs. However, the molecular mechanisms remain elusive. This study sought to determine PC targeted genes and the expression of select PC targeted genes in OA menisci to test hypothesis that PC exerts its disease modifying activity in part by reversing abnormal expressions of genes involved in OA. We found that PC downregulated the expression of numerous genes classified in immune response, inflammatory response, and angiogenesis, including chemokine (C-C motif) ligand 5, Fc fragment of IgG, low affinity IIIb receptor (FCGR3B), and leukocyte immunoglobulin-like receptor, subfamily B member 3 (LILRB3). In contrast, PC upregulated the expression of many genes classified in skeletal development, including collagen type II alpha1, fibroblast growth factor receptor 3 (FGFR3), and SRY- (sex determining region Y-) box 9 (SOX-9). Immunohistochemical examinations revealed higher levels of FCGR3B and LILRB3 and lower level of SOX-9 in OA menisci. These findings indicate that OA is a disease associated with immune system activation and decreased expression of SOX-9 gene in OA menisci. PC exerts its disease modifying activity on OA, at least in part, by targeting immune system activation and the production of extracellular matrix and selecting chondroprotective proteins.

## 1. Introduction

Osteoarthritis (OA) is one of the most prevalent causes of disability in the aging population and has enormous economic and social consequences. However, existing nonsurgical treatment options only provide symptomatic relief but have no effect on the progression of the disease. The lack of progress in the development of structural disease-modifying drugs for OA therapy is largely due to our limited understanding of the pathogenesis of OA and insufficient knowledge regarding the molecular targets or key OA disease genes for therapeutic intervention.

OA is not merely an articular cartilage disease, but a disease of the whole joint. An important local factor to the health of the knee joints is the structural integrity and biochemical properties of the knee meniscus. Knee meniscus is a specialized tissue that plays a vital role in load transmission, shock absorption, and joint stability. In recent years there has been a dramatic advance in our understanding of the integral role of the meniscus for the knee functions and the consequences of meniscal abnormality in cartilage degeneration. Studies found that meniscal degeneration is a general feature of OA [1, 2]; meniscal lesions at baseline were more common in the knees that developed OA than in the knees that did not develop OA [3] and that OA meniscal cells displayed a distinct gene expression profile different from normal meniscal cells [4]. These findings indicate that meniscal changes or abnormalities are involved in the OA disease process. The involvement of meniscal changes or abnormalities in the OA disease process has also been highlighted by recent findings that meniscal extrusion, vascular penetration (angiogenesis), and calcification are associated with cartilage degeneration and subchondral lesions in OA [5–7]. Meniscal abnormalities such as meniscal degeneration, inflammation, and angiogenesis may represent as new targets for the development of disease-modifying drugs for OA therapy, especially for a subgroup of OA patients who develop severe meniscal lesions before developing severe cartilage degeneration [8, 9].

Phosphocitrate (PC), a potent calcification inhibitor, is a naturally occurring compound originally identified in rat liver mitochondrial extract [10, 11]. PC prevented soft tissue calcification and inhibited calcium crystal-induced mitogenesis, crystal-induced expression of matrix metalloproteinases (MMPs), and crystal-induced cell death [12–15]. In Hartley guinea pig model of crystal-associated OA, PC inhibited meniscal calcification and reduced the severity of cartilage degeneration [16]. These findings provide support for the notion that calcification inhibitors are potentially disease modifying drugs for crystal-associated OA therapy. It is believed that PC exerts its disease modifying activity by inhibiting the formation of articular calcium crystals and the detrimental interaction between the crystals and cells [17]. However, two studies found that bisphosphonates, which are also potent calcification inhibitors, failed to inhibit cartilage degeneration in animal models of OA, including the Hartley guinea pig model of crystal-associated OA [18, 19], raising doubts as to whether calcification inhibitors are potentially disease-modifying drugs for OA therapy. An alternative mechanism underlying the disease modifying activity of PC may be present.

We previously reported that PC downregulated the expression of many genes classified in inflammatory response and angiogenesis in OA fibroblast-like synoviocytes (FLSs) and OA meniscal cells in the absence of calcium crystals [20, 21]. These findings suggest that the molecular mechanism underlying the disease-modifying activity of PC is more complicated than originally thought. In this study, we sought to further investigate the gene expression-modulating activity of PC and determine the expressions of select PC-targeted genes in menisci derived from OA patients. The hypothesis to be tested is that PC exerts its disease modifying activity, at least in part, by modulating the abnormal expressions of genes involved in the OA disease process. The information gained is not only important for a better understanding of the molecular mechanisms underlying the disease modifying activity of PC but may also valuable for the identification of disease candidate genes involved in the OA disease process.

## 2. Materials and Methods

### 2.1. Materials

Dulbecco's modified eagle medium (DMEM), fetal bovine serum (FBS), stock antibiotic/antimycotic mixture are products of Invitrogen (Carlsbad, CA, USA). Superfrost-Plus microscope slides and neutral buffered formalin (10%) were obtained from Allegiance Inc. (McGaw Park, IL, USA). PC was prepared as described [22]. Antibodies specific to Fc fragment of IgG, low affinity IIIb receptor (FCGR3B), SRY (sex determining region Y)-box 9 (Sox-9), and fibroblast growth factor receptor 3 (FGFR-3) were obtained from Santa Cruz Biotechnology (Dallas, TX, USA). Antibody specific to leukocyte immunoglobulin-like receptor, subfamily B member 3 (LILRB3) was obtained from Lifespan Biosciences (Seattle, WA, USA).

### 2.2. Meniscal Explant Culture and RNA Extraction

OA meniscal tissue specimens were minced into small pieces and cultured in a six well-cluster plate (350 mg per well) at 37°C in DMEM containing 1% FBS and 0.5% antibiotic/antimycotic solution. Twenty-four hours later, the medium in the top three wells was replaced with DMEM containing 1% FBS and 1 mM of PC and the medium in the bottom three wells was replaced with DMEM containing 1% FBS without PC as control. Every three days, the medium was changed. On the eighth day, the medium was changed again. Twenty-four hours later, the meniscal explants were collected, snap-freezed, and stored in −70°C freezer until use. Total RNA was extracted from these snap-freezed meniscal explants using Trizol reagent (Invitrogen, Carlsbad, CA, USA) and purified using Oligotex kit (Qiagen, Valencia, CA, USA). These RNA samples were used for microarray analysis.

Meniscal tissue specimens were collected with the approval of the authors' Institutional Review Board from end-stage OA patients undergoing knee joint replacement surgery and osteosarcoma patients undergoing lower limb amputation surgery at Carolinas Medical Center. The need for informed consent was waived because these meniscal specimens were surgical waste and no private patient information was collected. Meniscal specimens were collected in sterilized containers filled with tissue culture medium and transported to the laboratory from operating room using an ice box.

### 2.3. Microarray

RNA samples extracted from two independent experiments were used for microarray analysis. Briefly, double stranded DNA was synthesized using SuperScript double stranded cDNA synthesis kit using these RNA samples (Invitrogen, San Diego, CA, USA). The DNA product was purified using GeneChip sample cleanup module (Affymetrix, Santa Clara, CA, USA). cRNA was synthesized and biotin labeled using BioArray high yield RNA transcript labeling kit (Enzo Life Sciences, Farmingdale, NY, USA). The cRNA product was purified using GeneChip sample cleanup module and subsequently chemically fragmented. The fragmented and biotinylated cRNA was hybridized to HG-U133_Plus_2 gene chip using Affymetrix Fluidics Station 400 (Affymetrix, Santa Clara, CA, USA). The fluorescent signal was quantified during two scans by Agilent Gene Array Scanner G2500A (Agilent Technologies, Palo Alto, CA) and GeneChip operating Software (Affymetrix, Santa Clara, CA, USA). Genesifter software (VizX Labs, Seattle, WA, USA) was used for the analysis of differential gene expression and gene ontology.

### 2.4. Real-Time RT-PCR

Briefly, cDNA was synthesized using TaqMan Reverse Transcription Reagents (Applied Biosystems, University Park, IL, USA) using the RNA samples described. Quantification of relative transcript levels for selected genes and the housekeeping gene glyceraldehyde 3-phosphate dehydrogenase (GAPDH) was performed using ABI7000 Real Time PCR system (Applied Biosystems, University Park, IL, USA). TaqMan Gene Expression assay (Applied Biosystems, University Park, IL, USA) was used. CDNA samples were amplified with an initial Taq DNA polymerase activation step at 95°C for 10 minutes, followed by 40 cycles of denaturation at 95°C for 15 seconds and annealing at 60°C for one minute. Fold change was calculated and the expression level of the genes to be examined was normalized to the expression level of GAPDH. RT-PCR experiment was performed in triplicates using the same RNA sample for the microarray analysis.

### 2.5. Immunohistochemistry

Medial meniscal specimens derived from 6 end-stage OA patients and 3 osteosarcoma patients were used for examination. These meniscal specimens were fixed in 10% neutral buffered formalin for twenty-four hours and transferred to 70% ethyl alcohol. A portion of 5 mm wide specimen was transversely excised from the middle part of meniscus, embedded in Paraplast Plus paraffin, and sectioned with a Leica RM2025 microtome (Nussloch, Germany) to obtain 4 *μ*m serial transverse sections [23]. Sections were examined with immunohistochemical staining using specific antibodies. Briefly, paraffin-embedded sections were deparaffinized with xylene and rehydrated with graded ethanol. Endogenous peroxidase activity was blocked by incubation of the sections with freshly prepared 3% H_2_O_2_ in deionized water for 5 minutes at room temperature. Nonspecific binding was blocked by incubation of the sections with 100 *μ*L of 10% normal horse serum diluted in base solution (4% BSA and 5% nonfat dry milk in PBS) for 20 minutes. These sections were incubatedwith a specific primary antibody (2 *μ*g/mL) for 1 hour, followed with the secondary reagent specific for each antibody for 30 minutes (Immpress reagent kit, Vector, Inc., Burlingame, CA). Negative control was performed using mouse IgG to replace the primary specific antibody. Slides were rinsed in phosphate buffered saline three times and visualized using 3,3′-diaminobenzidine for 5 minutes. Slides were then counterstained with light green, dehydrated, and mounted with resinous mounting media. These immunostainings were graded on a scale of 0–3, where 0 = very weak staining; 1 = weak staining; 2 = moderate staining; 3 = strong staining.

### 2.6. Statistical Analysis

The difference between the immunostaining grades of the OA meniscal group and control group was analyzed using the Wilcoxon rank-sum test. *P* values less than 0.05 were considered significant. Statistical analysis was performed using the statistical analysis tool in the Sigma Plot software, version 12 (Systat software Inc., San Jose, CA).

## 3. Results

### 3.1. Effect of PC on Gene Expressions

Microarrayanalysis revealed that of the more than 50,000 transcripts examined, 2561 transcripts displayed significant differential expressions (more than 2.0 fold) in PC-treated OA meniscal explants via untreated OA meniscal explants. A total of 1430 transcripts displayed decreased expressions and 1131 transcripts displayed increased expressions. The genes that fell into specific biological processes previously implicated in OA or suspected to have a role in OA are listed in Tables 1, 2, and 3.

As shown in Table 1, the expressions of numerous genes classified in the immune response were downregulated by PC. Of the 120 differentially-expressed genes classified in the immune response, the expressions of 106 genes, including many genes encoding chemokines and cytokines, such as chemokine (C-C motif) ligand 20 (CCL20, −103.53 fold change), chemokine (C-C motif) ligand 5 (CCL5, −3.54 fold change), chemokine (C-C motif) receptor 5 (CCR5, −2.03 fold change), chemokine (C-X-C motif) ligand 3 (CXCL3, −10.03 fold), interleukin 6 (IL-6, −32.07 fold change), interleukin 7 receptor (IL-7R. −3.31 fold change), IL-8 (−8.17 fold change), IL-23, alpha subunit (IL23A, −5.46 fold change), and IL-1 beta (IL-1*β*, −2.28 fold change) genes, were downregulated by PC.

The genes downregulated by PC also included many Fc fragments of IgG receptors (FCGRs), leukocyte immunoglobulin-like receptors (LILRs), toll-like receptors (TLRs), and major histocompatibility complex (MHC) class II molecules genes, such as FCGR3B (−22.56 fold change), FCGR2B (−7.13 fold change), LILRB3 (−4.23 fold change), LILRB2 (−4.32 fold change), LILRB1 (−4.33 fold change), TLR8 (−5.55 fold change), TLR7 (−3.34 fold change), MHC class II, DP alpha 1 (HLA-DPA1, −2.83 fold change), MHC class II, DQ alpha 1 (HLA-DQA1, −3.39 fold), MHC class II, DR beta 1 (HLA-DRB1, −2.72 fold change), and MHC class II, DR beta 4 genes (HLA-DRB4, −2.81 fold change).

The expressions of many genes classified in inflammatory response and angiogenesis were also downregulated by PC. As shown in Table 2, of the 73 differentially-expressed genes classified in inflammatory response, the expressions of 64 genes, including prostaglandin-endoperoxide synthase 2 (PTGS2/Cox-2, −34.01 fold change), S100 calcium binding protein A8 (S100A8, −16.53 fold change), complement factor D (CFD, −4.99 fold change), and allograft inflammatory factor 1 (AIF1, −2.76 fold change) genes, were downregulated by PC. Of the 51 differentially-expressed genes classified in angiogenesis, the expressions of 31 genes, including brain-specific angiogenesis inhibitor 3 (BAI3, −33.48 fold change), angiopoietin-like 4 (ANGPTL4, −11.43 fold change), and vascular endothelial growth factor A genes (VEGFA, −7.91 fold change), were downregulated by PC.

In contrast, the expressions of many genes classified in skeletal development, steroid biosynthetic process, and DNA repair were upregulated by PC. As shown in Table 3, of the 26 differentially-expressed genes classified in skeletal development, the expressions of 17 genes, including collagen type II, alpha 1 (COL2A1, 21.29 fold change), collagen type XI, alpha 1 (COL11A1, 9.86 fold change), aggrecan (ACAN, 9.17 fold change), FGFR3 (2.40 fold change), FGF18 (2.33 fold change), and SOX-9 (2.00 fold change) genes, were upregulated by PC. Of the 16 differentially expressed genes classified in steroid biosynthetic process, the expressions of 11 genes, including squalene epoxidase (SQLE, 3.81 fold change) and steroid-5-alpha-reductase and alpha polypeptide 1 (SRD5A1, 3.75 fold change) genes, were upregulated by PC. Of the 7 differentially expressed genes classified in DNA repair, the expressions of 5 genes, including topoisomerase (DNA) II alpha 170 kDa (TOP2A, 3.75 fold change) and RAD52 homolog (RAD52B, 2.83 fold change) genes, were upregulated by PC.

We performed another independent microarray analysis using RNA samples extracted from meniscal explants derived from a different OA patient. Results from the two microarray analyses for select genes were listed in Table 4. As shown, the results from the two microarray analyses were consistent. We also examined the expressions of selected genes using real-time quantitative RT-PCR. The results from the real-time RT-PCR for the genes examined were consistent with the results from the microarray analyses (Table 4).

### 3.2. Immunohistochemical Staining

**Figure 1:**
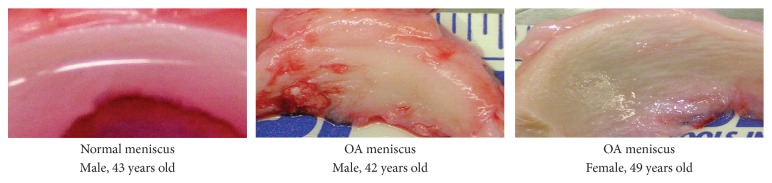
Images of normal and OA menisci. Meniscus derived from a male osteosarcoma patient, a male OA patient and a female OA patient. Menisci derived from OA patients exhibited discoloration and rough surface, with clear signs of degeneration whereas the normal control menisci had a smooth and glistening surface, with no signs of degeneration.

To investigate whether PC-targeted genes were associated with OA, we decided to examine the expressions of selected PC-targeted genes in OA and normal menisci. Medial menisci derived from 6 end-stage OA patients and 3 osteosarcoma patients were used for the examinations. Representative images of the normal meniscus and OA menisci are shown in Figure 1.

The expressions of genes we selected for immunohistochemical examinations included FCGR3B, LILRB3, FGFR3 and SOX-9. FCGR3B and LILRB3 are two genes classified in the immune and inflammatory responses. FGFR3 and SOX-9 were two genes classified in the skeletal development. We first tested the antibodies specific to these proteins using human tissues known for their expression before using these antibodies to examine the meniscal tissues.

**Figure 2:**
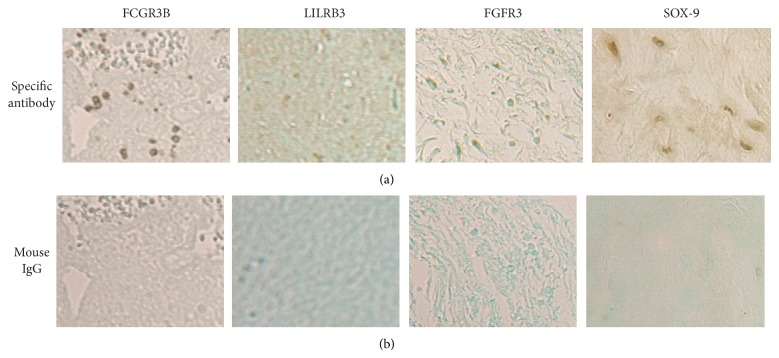
(a) Positive FCGR3B immunostaining in human tonsil tissue, positive LILRB3 immunostaining in human tonsil tissue, positive FGFR3 immunostaining in human skin tissue, and positive SOX-9 immunostaining in human cartilage tissue. (b) Negative immunostaining of these proteins when primary antibodies were replaced by mouse IgG.

As shown in Figure 2, positive immunostainings were observed when these primary antibodies specific to these proteins were used whereas no immunostainings were observed when these primary antibodies were replaced by mouse IgG, confirming that these antibodies could be used to examine the expressions of FCGR3B, LILRB3, FGFR3, and SOX-9 in human tissues. We then used these antibodies to examine medial meniscal specimens derived from 6 end-stage OA patients (diseased tissues) and 3 osteosarcoma patients (control tissues). Representative images of FCGR3B immunostaining are provided in Figure 2(a).

**Figure 3:**
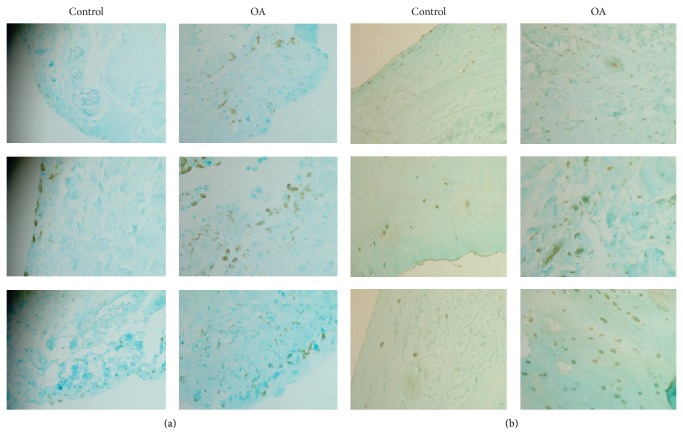
Representative images of FCGR3B and LILR3B immunostaining (magnification 10x). (a) FCGR3B immunostaining of normal menisci and OA menisci. (b) LILRB3 immunostaining of normal menisci and OA menisci.

As shown, FCGR3B protein was detected in the surface zone of normal menisci. In contrast, FCGR3B was detected in both the surface and middle zones of OA menisci. It was clear that OA menisci contained more FCGR3B immunostaining-positive cells than the normal menisci, suggesting infiltration of inflammatory cells within the OA menisci. Representative images of LILRB3 immunostaining are provided in Figure 3(b). As shown, LILRB3 protein was detected in all 3 zones (surface, middle, and deep zones) of the normal and OA menisci. OA menisci not only contained more LILRB3 immunostaining-positive cells but also displayed more intensified LILRB3 immunostaining compared to the normal menisci.

**Figure 4:**
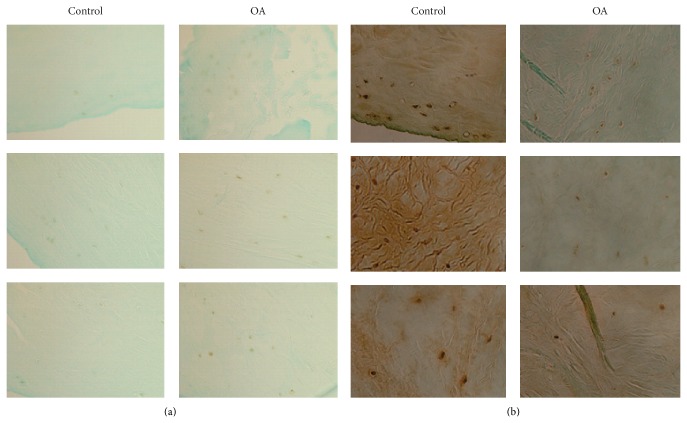
Representative images of FGFR3 and SOX-9 immunostaining (magnification 10x). (a) FGFR3 immunostaining of normal menisci and OA menisci. (b) SOX-9 immunostaining of normal menisci and OA menisci.

Representative images of FGFR3 and SOX-9 immunostaining are provided in Figure 4. As shown in Figure 4(a), few FGFR3 immunostaining-positive cells were detected in the normal menisci and only a small number of FGFR3 immunostaining-positive cells were detected in the OA menisci. In contrast, strong SOX-9 immunostaining was detected in the normal menisci and the intensity of SOX-9 immunostaining and number of SOX-9 immunostaining-positive cells were decreased significantly in the OA menisci compared to the normal menisci (Figure 4(b)).

The immunostainings of FCGR3B, LILRB3, FGFR3, and SOX-9 were graded according to the scale described in Methods. The results along with the demographic patient information are listed in Table 5. As shown, the mean grades of FCGR3B, LILRB3, FGFR3, and SOX-9 immunostainings for the normal menisci were 0.33, 1.33, 0.00, and 3.00, respectively, and the mean grades of FCGR3B, LILRB3, FGFR3, and SOX-9 immunostaining for the OA menisci were 2.00, 2.67, 1.17, and 1.50, respectively. The difference between the mean grades of FCGR3B, LILRB3, FGFR3, and SOX-9 immunostaining of the OA menisci and the normal menisci were statistically significant (*P* = 0.023, 0.010, 0.002, and 0.024, resp.).

## 4. Discussion

There is increasing evidence indicating the involvement of immune system in OA. The expressions of several TLRs genes, which play a key role in innate immune system, were increased in OA cartilage and correlated with the severity of cartilage degeneration [24, 25]. The expressions of several MHC class II genes were increased in degenerative menisci of older patients and OA meniscal cells compared to younger patients and normal meniscal cells [4, 26]. In this study, we demonstrated that PC, which inhibited cartilage degeneration in Hartley guinea pigs [16], downregulated the expression of numerous genes classified in the immune response, including many TLRs genes (Table 1, such as TLR-4 and TLR-8), MHC class II genes (such as HLA-DPA1, HLA-DRA, and HLA-DRB1), FCGRs genes (such as FCGR2B and FCGR3B), and LILRs genes (such as LILRA2, LILRB1, and LILRB3). These findings suggest that PC may be capable of reversing the abnormal expressions of many genes involved in immune system activation in OA menisci and cartilage.

Studies found that the expression of FCGRs, which help to bridge the adaptive and innate immune responses, and the expression of LILRs, which exert influence on signaling pathways of both innate and adaptive immune systems, were increased in inflammatory arthritis such as rheumatoid arthritis (RA) [27–30]. In addition, studies found that the numbers of FCGRs- and LILRs-positive immune cells were decreased in RA patients who responded to treatment with anti-rheumatic drugs [31, 32]. These previous findings indicate that abnormal expressions of FCGRs and LILRs are associated with inflammatory arthritis. In this study, we demonstrated that the expressions of FCGR3B and LILRB3 genes were increased in OA menisci and that their expressions in OA meniscal explant culture were inhibited by PC. These findings indicate that abnormal expressions of FCGRs and LILRs are also associated with OA. PC exerts its disease-modifying activity on OA, at least in part, by targeting abnormal immune system activation in OA.

Inflammation and angiogenesis are closely integrated processes in OA [33, 34]. A study demonstrated that inhibition of inflammation and angiogenesis reduced pain and retard joint damage in a rat model of OA [35]. In our study, we demonstrated that PC downregulated the expression of numerous genes classified in the inflammatory response and angiogenesis, including CCL5, CCR5, IL-8, IL-7R, IL-6, IL-1*β*, PTGS2/Cox-2, S100A8, ANGPTL4, and VEGFA. It is worth noting that abnormal expressions of these genes are associated with either OA or RA. For example, the protein levels of CCL5, IL-6, IL-8, IL-1*β*, S100A8, ANGPTL4, and VEGFA were increased in chondrocytes, cartilage, synovium, or synovial fluid derived from OA patients, which in turn stimulated the expression of MMPs [36–44]; the expression of IL-7R was elevated in RA FLSs and blockade of IL-7R reduced joint inflammation and cartilage destruction [45, 46]; PTGS2/Cox-2 is a key molecular target for the management of arthritis pain [47]. These findings together suggest that PC exerts its disease-modifying activity on OA, at least in part, by targeting abnormal inflammatory response and angiogenesis in OA. These findings also suggest that abnormal inflammatory response and angiogenesis in the menisci may be new target for OA intervention.

PC upregulated the expressions of many genes classified in skeletal development, including putative chondroprotective proteins FGFR3 and SOX-9 [48, 49]. To identify which proteins might be involved in the OA disease process, we examined the protein levels of FGFR3 and SOX-9 in normal and OA menisci. We found that FGFR3 protein was barely detected in the normal menisci and was slightly increased in OA menisci, suggesting that FGFR3 gene is unlikely a key OA disease candidate gene. In contrast, the protein level of SOX-9 was very high in the normal menisci but was significantly decreased in the OA menisci. This finding is consistent with the previous findings that the expression of SOX-9 gene was significantly decreased in OA articular cartilage and chondrocytes [50–52]. Taken together, it suggests that SOX-9 may be an OA disease candidate gene and that PC exerts its disease modifying activity on OA in part by reversing the abnormal expression of SOX-9 in OA menisci or cartilage. Study with SOX-9 knok-in or knock-out using an animal model of OA may provide more clues about the role of SOX-9 in OA.

A recent study reported that hundreds of genes were differentially expressed in degenerative menisci derived from older patients and younger patients [26]. The genes displayed higher expressions in the degenerative menisci derived from older patients compared to younger patients included HLA-DRB1 (15.01 fold change), FCER1A (4.15 fold change), and IL-7R (2.83 fold change) [26]. These findings are consistent with our findings and indicate that immune system activation occurs in the degenerative menisci. These findings, together with our findings, also suggest that increased expressions of HLA-DRB and IL-7R is likely a phenomenon associated with both the normal meniscal aging process and OA disease process whereas the increased expression of FCER1A is a phenomenon only associated with the normal meniscal aging process.

The genes displayed lower expression in the degenerative menisci derived from older patients included COL2A1 (−10.38 fold change) and FGFR3 (−4.65 fold change) [26]. These findings, together with our findings [23], indicate that the decreased expression of COL2A1 is likely a phenomenon associated with both the normal meniscal aging process and OA disease process whereas the decreased expression of FGFR3 is a phenomenon associated with the meniscal aging process. Our findings presented in this study demonstrate that PC affects the expressions of many genes involved in both OA disease process and meniscal aging process in the absence of calcium crystals. This suggests that PC is not only potentially a disease-modifying drug for calcification-induced OA therapy but also potentially a disease-modifying drug for noncalcification-induced arthritis therapy such as posttraumatic OA.

## 5. Conclusions

OA is a disease associated with immune system activation and decreased expression of chondroprotective protein SOX-9. PC exerts its disease-modifying activity on OA, at least in part, by suppressing immune system activation and stimulating the production of extracellular cellular matrix proteins and chondroprotective proteins. PC is potentially a disease-modifying drug for noncalcification-induced arthritis therapy.

## Figures and Tables

**Table 1:** Genes classified in the immune response.

Biological process	Gene name	Gene ID	Differ expre^*^	Description
Immune response				
	CCL20	NM_004591	−103.53	Chemokine (C-C motif) ligand 20
	CCL5	NM_002985	−3.54	Chemokine (C-C motif) ligand 5
	CCR5	NM_000579	−2.03	Chemokine (C-C motif) receptor 5
	CXCL3	NM_002090	−10.03	Chemokine (C-X-C motif) ligand 3
	CXCL5	AK026546	−4.49	Chemokine (C-X-C motif) ligand 5
	CXCL2	M57731	−2.94	Chemokine (C-X-C motif) ligand 2
	CXCL1	NM_001511	−3.01	Chemokine (C-X-C motif) ligand 1
	CXCL9	NM_002416	−2.65	Chemokine (C-X-C motif) ligand 9
	CXCL13	NM_006419	−2.62	Chemokine (C-X-C motif) ligand 13 (B-cell chemoattractant)
	CXCR4	AF348491	−2.63	Chemokine (C-X-C motif) receptor 4
	IGKC	BC005332	−37.44	Netrin 2-like (chicken)
	IL6	NM_000600	−32.07	Interleukin 6 (interferon, beta 2)
	IL8	NM_000584	−8.17	Interleukin 8
	IL23A	M15564	−7.89	Enhancer of polycomb homolog 1 (Drosophila)
	IL24	NM_006850	−3.73	Interleukin 24
	IL15	NM_000585	−3.70	Interleukin 15
	IL7R	BE217880	−3.31	Interleukin 7 receptor
	IL1B	NM_000576	−2.28	Interleukin 1, beta
	IL1RN	BE563442	−2.80	Interleukin 1 receptor antagonist
	MS4A2	NM_000139	−28.91	Membrane-spanning 4-domains, subfamily A, member 2
	FCGR3B	NM_000570	−22.56	Fc fragment of IgG, low affinity IIIb, receptor (CD16b)
	FCGR2B	U90940	−7.13	Fc fragment of IgG, low affinity IIc, receptor for (CD32)
	FCGR1B	L03419	−3.30	Fc fragment of IgG, high affinity Ib, receptor (CD64)
	FCER1A	BC005912	−5.19	Fc fragment of IgE, high affinity I, receptor for; alpha polypeptide
	HLA-C	AW575927	−22.41	Major histocompatibility complex, class I, C
	HLA-F	BE138825	−2.22	Major histocompatibility complex, class I, F
	AQP9	NM_020980	−17.49	Aquaporin 9
	GZMA	NM_006144	−14.63	Granzyme A
	IGHG1	M87789	−14.43	Immunoglobulin heavy constant gamma 1
	LBP	M35533	−11.36	Lipopolysaccharide binding protein
	EREG	NM_001432	−8.60	Epiregulin
	CD86	NM_006889	−7.11	CD86 molecule
	CD74	M28590	−2.76	CD74 molecule
	CD40	NM_001250	−3.68	CD40 molecule, TNF receptor superfamily member 5
	CD1D	NM_001766	−5.17	CD1d molecule
	CD8A	AW006735	−4.99	CD8a molecule
	CD14	NM_000591	−3.25	CD14 molecule
	CD209	AF290886	−2.50	CD209 molecule
	KYNU	BC000879	−6.69	Kynureninase (L-kynurenine hydrolase)
	PTPRC	NM_002838	−5.88	Protein tyrosine phosphatase, receptor type, C
	TLR8	AW872374	−5.55	Toll-like receptor 8
	TLR7	NM_016562	−3.34	Toll-like receptor 7
	TLR5	AF051151	−3.00	Toll-like receptor 5
	TLR4	NM_003266	−2.40	Toll-like receptor 4
	TLR2	NM_003264	−2.20	Toll-like receptor 2
	TLR1	AL050262	−2.66	Toll-like receptor 1
	SLC11A1	L32185	−5.41	Solute carrier family 11, member 1
	CFD	NM_001928	−4.99	Complement factor D (adipsin)
	CFI	BC020718	−3.37	Complement factor I
	CFB	NM_001710	−2.39	Complement factor B
	C3	NM_000064	−2.32	Complement component 3
	CR1	AI052659	−2.24	Complement component (3b/4b) receptor 1 (Knops blood group)
	C1QA	NM_015991	−2.20	Complement component 1, q subcomponent, A chain
	C1QB	NM_000491	−2.10	Complement component 1, q subcomponent, B chain
	C1RL	NM_016546	2.01	Complement component 1, r subcomponent-like
	FYB	BF679849	−4.64	FYN binding protein (FYB-120/130)
	TREM1	NM_018643	−4.52	Triggering receptor expressed on myeloid cells 1
	BMP6	NM_001718	−4.48	Bone morphogenetic protein 6
	INPP5D	U53470	−4.47	Inositol polyphosphate-5-phosphatase, 145 kDa
	LILRB1	NM_006669	−4.33	Leukocyte immunoglobulin-like receptor, subfamily B, member 1
	LILRB2	AF004231	−4.32	Leukocyte immunoglobulin-like receptor, subfamily B, member 2
	LILRB3	AF009634	−4.23	leukocyte immunoglobulin-like receptor, subfamily B, member 3
	LILRB4	U82979	−2.06	Leukocyte immunoglobulin-like receptor, subfamily B, member 4
	LILRB5	NM_006840	−2.31	Leukocyte immunoglobulin-like receptor, subfamily B, member 5
	LILRA2	NM_006866	−2.15	Leukocyte immunoglobulin-like receptor, subfamily A, member 2
	ZEB1	NM_030751	−4.12	Zinc finger E-box binding homeobox 1
	PLA2G7	M80436	−3.95	Platelet-activating factor receptor
	MASP1	AI274095	−3.67	Mannan-binding lectin serine peptidase 1
	EBI2	NM_004951	−3.59	Epstein-Barr virus induced gene 2
	NOD2	NM_022162	−3.57	Nucleotide-binding oligomerization domain containing 2
	LAIR1	NM_021708	−3.54	Leukocyte-associated immunoglobulin-like receptor 1
	HLA-DQA1	BG397856	−3.38	Major histocompatibility complex, class II, DQ alpha 1
	HLA-DQB1	M17955	−2.99	Major histocompatibility complex, class II, DQ beta 1
	HLA-DRA	M60333	−3.22	Major histocompatibility complex, class II, DR alpha
	HLA-DPA1	M27487	−2.83	Major histocompatibility complex, class II, DP alpha 1
	HLA-DPB1	NM_002121	−2.15	Major histocompatibility complex, class II, DP beta 1
	HLA-DRB4	BC005312	−2.81	Major histocompatibility complex, class II, DR beta 4
	HLA-DRB1	AJ297586	−2.72	Major histocompatibility complex, class II, DR beta 3
	HLA-DMB	NM_002118	−2.68	Major histocompatibility complex, class II, DM beta
	HLA-DMA	X76775	−2.19	major histocompatibility complex, class II, DM alpha
	LIF	NM_002309	−3.16	Leukemia inhibitory factor (cholinergic differentiation factor)
	NCF4	NM_000631	−2.88	Neutrophil cytosolic factor 4, 40 kDa
	PAG1	BF589359	−2.86	Phosphoprotein associated with glycosphingolipid microdomains 1
	FYN	AK090692	−2.85	FYN oncogene related to SRC, FGR, YES
	TREM2	NM_018965	−2.84	Triggering receptor expressed on myeloid cells 2
	BST2	NM_004335	−2.84	Bone marrow stromal cell antigen 2
	CTSG	NM_001911	−2.78	Cathepsin G
	CTSS	AK024855	−2.60	Cathepsin S
	MS4A1	AW474852	−2.74	Membrane-spanning 4-domains, subfamily A, member 1
	NCF2	BC001606	−2.70	Neutrophil cytosolic factor 2
	GPR65	NM_003608	−2.68	G protein-coupled receptor 65
	GBP4	BG260886	−2.59	Guanylate binding protein 4
	VAV1	NM_005428	−2.53	Vav 1 guanine nucleotide exchange factor
	LCK	NM_005356	−2.49	Lymphocyte-specific protein tyrosine kinase
	SYK	NM_003177	−2.42	Spleen tyrosine kinase
	LY86	NM_004271	−2.36	Lymphocyte antigen 86
	TNFSF10	U57059	−2.36	Tumor necrosis factor (ligand) superfamily, member 10
	TNFSF13B	AF134715	−2.29	Tumor necrosis factor (ligand) superfamily, member 13b
	IRF8	AI073984	−2.36	Interferon regulatory factor 8
	RELB	NM_006509	−2.33	V-rel reticuloendotheliosis viral oncogene homolog B
	SMAD6	AI628464	−2.25	SMAD family member 6
	MBP	N37023	−2.24	Myelin basic protein
	BCL6	S67779	−2.17	B-cell CLL/lymphoma 6 (zinc finger protein 51)
	IGKC	BG485135	−2.12	Netrin 2-like (chicken)
	CLEC7A	AF313468	−2.12	C-type lectin domain family 7, member A
	LCP2	AI123251	−2.05	Lymphocyte cytosolic protein 2
	IL31RA	AI123586	6.61	Interleukin 31 receptor A
	C4BPA	NM_000715	3.34	Complement component 4 binding protein, alpha
	ULBP2	AA831769	3.07	UL16 binding protein 2
	CLEC4E	NM_014358	2.61	C-type lectin domain family 4, member E
	TNFSF9	NM_003811	2.43	Tumor necrosis factor (ligand) superfamily, member 9
	C7	NM_000587	2.36	Complement component 7
	TGFB2	NM_003238	2.36	Transforming growth factor, beta 2
	FAS	X83493	2.36	Fas (TNF receptor superfamily, member 6)
	LAG3	NM_002286	2.26	Lymphocyte-activation gene 3
	IL27RA	NM_004843	2.20	Interleukin 27 receptor, alpha
	CD276	NM_025240	2.19	CD276 molecule
	IL26	NM_018402	2.03	Interleukin 26
	OAS1	NM_016816	2.02	2,5-oligoadenylate synthetase 1, 40/46 kDa

^*^Negative number indicates decreased expression and positive number indicates increased expression (fold change) in PC-treated OA meniscal explants compared with untreated OA meniscal explants.

**Table 2:** Genes classified in inflammatory response and angiogenesis.

Biological process	Gene name	Gene ID	Differ expre (fold)^*^	Description
Inflammatory response				
	CCL20	NM_004591	−103.53	Chemokine (C-C motif) ligand 20
	CCL5	NM_002985	−3.54	Chemokine (C-C motif) ligand 5
	CCR5	NM_000579	−2.03	Chemokine (C-C motif) receptor 5
	CXCL3	NM_002090	−10.03	Chemokine (C-X-C motif) ligand 3
	CXCL2	M57731	−2.94	Chemokine (C-X-C motif) ligand 2
	CXCL1	NM_001511	−3.01	Chemokine (C-X-C motif) ligand 1
	CXCL9	NM_002416	−2.65	Chemokine (C-X-C motif) ligand 9
	CXCL13	NM_006419	−2.62	Chemokine (C-X-C motif) ligand 13 (B-cell chemoattractant)
	CXCR4	AF348491	−2.63	Chemokine (C-X-C motif) receptor 4
	PTGS2	AY151286	−34.01	Prostaglandin-endoperoxide synthase 2
	IL6	NM_000600	−32.07	Interleukin 6 (interferon, beta 2)
	IL23A	AF043179	−16.44	Enhancer of polycomb homolog 1 (Drosophila)
	IL8	NM_000584	−8.17	Interleukin 8
	IL1B	NM_000576	−2.28	Interleukin 1, beta
	IL1RN	BE563442	−2.80	Interleukin 1 receptor antagonist
	S100A8	NM_002964	−16.53	S100 calcium binding protein A8
	S100A9	NM_002965	−2.79	S100 calcium binding protein A9
	LBP	M35533	−11.36	Lipopolysaccharide binding protein
	APOE	NM_000041	−9.23	Apolipoprotein E
	FABP4	AI766029	−6.98	Fatty acid binding protein 4, adipocyte
	LYZ	U25677	−6.37	Lysozyme (renal amyloidosis)
	ITIH4	AI004137	−6.33	Inter-alpha (globulin) inhibitor H4
	BDKRB2	NM_000623	−6.01	Bradykinin receptor B2
	TLR8	AW872374	−5.55	Toll-like receptor 8
	TLR7	NM_016562	−3.34	Toll-like receptor 7
	TLR5	AF051151	−3.00	Toll-like receptor 5
	TLR4	NM_003266	−2.40	Toll-like receptor 4
	TLR2	NM_003264	−2.20	Toll-like receptor 2
	TLR1	AL050262	−2.66	Toll-like receptor 1
	AOX1	AB046692	−5.34	Aldehyde oxidase 1
	FCER1A	BC005912	−5.19	Fc fragment of IgE, high affinity I, receptor for; alpha polypeptide
	CFD	NM_001928	−4.99	Complement factor D (adipsin)
	CFI	BC020718	−3.37	Complement factor I
	CFB	NM_001710	−2.39	Complement factor B
	C3	NM_000064	−2.32	Complement component 3
	CR1	AI052659	−2.24	Complement component (3b/4b) receptor 1 (Knops blood group)
	C1RL	NM_016546	−2.01	Complement component 1, r subcomponent-like
	C1QA	NM_015991	−2.20	Complement component 1, q subcomponent, A chain
	C1QB	NM_000491	−2.10	Complement component 1, q subcomponent, B chain
	AOAH	NM_001637	−4.89	Acyloxyacyl hydrolase (neutrophil)
	AGT	NM_000029	−4.66	Angiotensinogen (serpin peptidase inhibitor, clade A, member 8)
	BMP6	NM_001718	−4.48	Bone morphogenetic protein 6
	PLA2G7	M80436	−3.95	Platelet-activating factor receptor
	FOS	BC004490	−3.95	V-fos FBJ murine osteosarcoma viral oncogene homolog
	CD40	NM_001250	−3.68	CD40 molecule, TNF receptor superfamily member 5
	CD163	NM_004244	−3.68	CD163 molecule
	CD14	NM_000591	−3.25	CD14 molecule
	MASP1	AI274095	−3.67	Mannan-binding lectin serine peptidase 1
	F11R	AF191495	−3.16	F11 receptor
	ITGB2	L78790	−2.98	Integrin, beta 2 (complement component 3 receptor 3 and 4 subunit)
	NLRC4	NM_021209	−2.92	NLR family, CARD domain containing 4
	AIF1	U19713	−2.76	Allograft inflammatory factor 1
	ALOX5	NM_000698	−2.71	Arachidonate 5-lipoxygenase
	NOD1	AF126484	−2.66	Nucleotide-binding oligomerization domain containing 1
	JMJD3	AI830331	−2.46	Jumonji domain containing 3, histone lysine demethylase
	SIGLEC1	NM_023068	−2.46	Sialic acid binding Ig-like lectin 1, sialoadhesin
	TNFAIP6	AW188198	−2.37	Tumor necrosis factor, alpha-induced protein 6
	LY86	NM_004271	−2.36	Lymphocyte antigen 86
	AOC3	NM_003734	−2.31	Amine oxidase, copper containing 3 (vascular adhesion protein 1)
	TNFRSF1B	NM_001066	−2.21	Tumor necrosis factor receptor superfamily, member 1B
	BCL6	S67779	−2.17	B-cell CLL/lymphoma 6 (zinc finger protein 51)
	CLEC7A	AF313468	−2.12	C-type lectin domain family 7, member A
	CDO1	NM_001801	−2.11	Cysteine dioxygenase, type I
	NFATC4	AI806528	−2.04	NF of activated T-cells, cytoplasmic, calcineurin-dependent 4
	SERPINA3	NM_001085	5.20	Serpin peptidase inhibitor, clade A, member 3
	SERPINA1	AF119873	3.58	Serpin peptidase inhibitor, clade A, member 1
	C4BPA	NM_000715	3.34	Complement component 4 binding protein, alpha
	FN1	AJ276395	3.03	Fibronectin 1
	B4GALT1	D29805	2.99	UDP-Gal:betaGlcNAc beta 1,4-galactosyltransferase, polypeptide 1
	GPR68	AI805006	2.40	G protein-coupled receptor 68
	C7	NM_000587	2.36	Complement component 7
	ANXA1	AU155094	2.23	Annexin A1
	KLKB1	NM_000892	2.21	Cytochrome P450, family 4, subfamily V, polypeptide 2
Angiogenesis				
	PTGS2	AY151286	−34.00	Prostaglandin-endoperoxide synthase 2
	BAI3	NM_001704	−33.48	Brain-specific angiogenesis inhibitor 3
	IL6	NM_000600	−32.08	Interleukin 6 (interferon, beta 2)
	IL8	NM_000584	−8.18	Interleukin 8
	IL1B	NM_000576	−2.28	Interleukin 1, beta
	SFRP1	AF017987	−17.55	Secreted frizzled-related protein 1
	ANGPTL4	AF169312	−11.43	Angiopoietin-like 4
	ANGPT2	BE501356	−2.56	Angiopoietin 2
	EREG	NM_001432	−8.60	Epiregulin
	VEGFA	M27281	−7.91	Vascular endothelial growth factor A
	VASH1	AU152507	−4.24	Vasohibin 1
	FLT1	U01134	−3.62	Fms-related tyrosine kinase 1
	PTPRB	AL080103	−3.48	Protein tyrosine phosphatase, receptor type, B
	FGF10	NM_004465	−3.34	Fibroblast growth factor 10
	LIF	NM_002309	−3.16	Leukemia inhibitory factor (cholinergic differentiation factor)
	BTG1	BC009050	−3.11	B-cell translocation gene 1, anti-proliferative
	DLL4	AB036931	−2.85	Delta-like 4 (Drosophila)
	SOX17	NM_022454	−2.45	SRY (sex determining region Y)-box 17
	EPAS1	NM_001430	−2.43	Endothelial PAS domain protein 1
	CTNNB1	AB062292	−2.35	Catenin (cadherin-associated protein), beta 1, 88 kDa
	TGFBR2	NM_003242	−2.35	Transforming growth factor, beta receptor II (70/80 kDa)
	TYMP	NM_001953	−2.34	Thymidine phosphorylase
	C3	NM_000064	−2.32	Complement component 3
	NRP1	AF280547	−2.19	Neuropilin 1
	NOTCH4	AI743713	−2.17	Notch homolog 4 (Drosophila)
	TSPAN12	AI056699	−2.16	Tetraspanin 12
	ADAM8	NM_001109	−2.15	ADAM metallopeptidase domain 8
	RHOB	AI263909	−2.12	Ras homolog gene family, member B
	HHEX	NM_001529	−2.12	Hematopoietically expressed homeobox
	THBS1	BF055462	−2.05	Thrombospondin 1
	KDR	NM_002253	−2.03	Kinase insert domain receptor (a type III receptor tyrosine kinase)
	GREM1	NM_013372	15.86	Gremlin 1, cysteine knot superfamily, homolog (*Xenopus laevis*)
	FGFR2	AB030078	4.49	Fibroblast growth factor receptor 2
	FGF1	X59065	2.39	fibroblast growth factor 1 (acidic)
	FGF18	AI798863	2.33	Fibroblast growth factor 18
	COL8A2	AI806793	4.46	Collagen, type VIII, alpha 2
	COL8A1	BE877796	3.30	Collagen, type VIII, alpha 1
	ARHGAP22	NM_021226	4.45	Rho GTPase activating protein 22
	TNFRSF12A	NM_016639	3.23	Tumor necrosis factor receptor superfamily, member 12A
	CSPG4	BE857703	3.14	Chondroitin sulfate proteoglycan 4
	WNT5B	NM_030775	3.10	Wingless-type MMTV integration site family, member 5B
	FN1	AJ276395	3.03	Fibronectin 1
	SFRP2	AF311912	3.00	Secreted frizzled-related protein 2
	B4GALT1	D29805	2.99	UDP-Gal:betaGlcNAc beta 1,4-galactosyltransferase, polypeptide 1
	ANGPT1	NM_001146	2.62	Angiopoietin 1
	MFGE8	BC003610	2.39	Milk fat globule-EGF factor 8 protein
	TGFB2	NM_003238	2.36	Transforming growth factor, beta 2
	CYR61	NM_001554	2.30	Cysteine-rich, angiogenic inducer, 61
	MEOX2	NM_005924	2.15	Mesenchyme homeobox 2
	PLXDC1	NM_020405	2.09	Plexin domain containing 1
	TBXA2R	NM_001060	2.09	Thromboxane A2 receptor

^*^Negative number indicates decreased expression and positive number indicates increased expression (fold change).

**Table 3:** Genes classified in skeletal development, steroid biosynthetic process, and DNA repair.

Biological process	Gene name	Gene ID	Differ expre (fold)^*^	Description
Skeletal development				
	COL2A1	X06268	21.29	Collagen, type II, alpha 1
	COL11A1	J04177	9.86	Collagen, type XI, alpha 1
	COL1A1	AI743621	2.59	Collagen, type I, alpha 1
	ACAN	NM_001135	9.17	Aggrecan
	POSTN	AW137148	5.57	Periostin, osteoblast specific factor
	PAX1	AA725078	4.73	Paired box 1
	FRZB	U91903	3.22	Frizzled-related protein
	MMP9	NM_004994	2.58	Matrix metallopeptidase 9
	GLI2	NM_030379	2.54	GLI-Kruppel family member GLI2
	FGFR3	NM_000142	2.40	Fibroblast growth factor receptor 3
	FGF18	AI798863	2.33	Fibroblast growth factor 18
	TGFB2	NM_003238	2.36	Transforming growth factor, beta 2
	TRPS1	AK000948	2.32	Trichorhinophalangeal syndrome I
	RUNX2	AW469546	2.24	Runt-related transcription factor 2
	PRELP	U41344	2.15	Proline/arginine-rich end leucine-rich repeat protein
	PAPSS2	AW299958	2.03	3-phosphoadenosine 5-phosphosulfate synthase 2
	SOX9	NM_000346	2.00	SRY (sex determining region Y)-box 9
	MEPE	NM_020203	−13.5	Matrix, extracellular phosphoglycoprotein with ASARM motif
	PTHLH	BC005961	−6.79	Parathyroid hormone-like hormone
	CHRDL2	AF332891	−6.20	Chordin-like 2
	COL10A	X98568	−5.40	collagen, type X, alpha 1 (Schmid metaphyseal chondrodysplasia)
	BMP6	NM_001718	−4.48	Bone morphogenetic protein 6
	GDF10	NM_004962	−2.99	Growth differentiation factor 10
	BMP8B	AA610122	−2.70	Bone morphogenetic protein 8b
	TGFBR2	NM_003242	−2.35	Transforming growth factor, beta receptor II (70/80 kDa)
	IGFBP4	NM_001552	−2.30	Insulin-like growth factor binding protein 4
Steroid biosynthetic process				
	SQLE	AA639705	3.81	Squalene epoxidase
	SRD5A1	NM_001047	3.75	Steroid-5-alpha-reductase, alpha polypeptide 1
	FDXR	NM_004110	3.26	Ferredoxin reductase
	HMGCS1	NM_002130	3.12	3-hydroxy-3-methylglutaryl-Coenzyme A synthase 1 (soluble)
	HMGCR	AL518627	2.15	3-hydroxy-3-methylglutaryl-Coenzyme A reductase
	LSS	D63807	3.04	Lanosterol synthase (2,3-oxidosqualene-lanosterol cyclase)
	DHCR24	NM_014762	2.66	24-dehydrocholesterol reductase
	DHCR7	NM_001360	2.57	7-dehydrocholesterol reductase
	OPRS1	NM_005866	2.62	Opioid receptor, sigma 1
	CYB5R2	NM_016229	2.37	Cytochrome b5 reductase 2
	SC5D	D85181	2.06	Sterol-C5-desaturase
	BMP6	NM_001718	−4.48	Bone morphogenetic protein 6
	CYP39A1	NM_016593	−2.21	Cytochrome P450, family 39, subfamily A, polypeptide 1
	HSD3B7	BC004929	−2.10	3 beta-hydroxysteroid dehydrogenase type 7
	ADM	NM_001124	−2.06	Adrenomedullin
	ABCG1	NM_004915	−2.04	ATP-binding cassette, sub-family G (WHITE), member 1
DNA repair				
	TOP2A	AU159942	3.75	Topoisomerase (DNA) II alpha 170 kDa
	RAD52B	AF125949	2.83	RAD52 homolog (*S. cerevisiae*)
	RAD54B	NM_012415	2.05	RAD54 homolog B (*S. cerevisiae*)
	TYMS	NM_001071	2.40	Thymidylate synthetase
	RFC5	BG260658	2.39	Replication factor C (activator 1) 5, 36.5 kDa
	SOD2	AL050388	−9.58	Superoxide dismutase 2, mitochondrial
	REV3L	NM_002912	−3.08	REV3-like, catalytic subunit of DNA polymerase zeta (yeast)

^*^Negative number indicates decreased expression and positive number indicates increased expression (fold change).

**Table 4:** Differential expression of selected genes.

Gene name	Gene ID	Differential expression^*^ microarray	Differential expression^**^ microarray	Differential expression^***^ real-time RT-PCR
CCL5	NM_002985	−3.54	−6.28	−3.69
CCR5	NM_000579	−2.03	−2.95	
FCGR3B	NM_000570	−6.49	−2.09	
FCGR2B	M31933	−3.45	−3.72	
IL6	NM_000600	−32.07	−1.85	
IL7R	BE217880	−3.31	−11.0	−2.85
IL8	NM_000584	−8.17	−2.25	
IL23A	M15564	−7.89	−8.98	
LILRB1	NM_006669	−4.33	−3.06	
LILRB3	AF009634	−4.23	−3.38	
TLR8	AW872374	−5.55	−3.55	
TLR7	NM_016562	−3.34	−2.54	
HLA-DRB1	AJ297586	−2.72	−3.41	−1.97
S100A8	NM_002964	−16.53	−9.95	
S100A9	NM_002965	−2.79	−4.70	
PTPRC	NM_002838	−5.88	−2.85	
SYK	NM_003177	−2.42	−2.99	
BMP6	NM_001718	−4.48	−3.00	
CPA3	NM_001870	−15.23	−9.37	
CPM	BE878495	−3.17	−2.74	
ADAMTS5	BI254089	−2.84	−1.98	−2.65
ADAM28	NM_021778	−14.27	−3.59	
MMP10	NM_002425	−2.41	−1.56	
MMP1	NM_002421	−2.15	−1.83	−2.01
FGFR3	NM_000142	2.40	1.52	
SOX-9	NM_000346	2.00	1.70	
POSTN	AW137148	5.57	2.76	
COL2A1	X06268	21.29	1.76	3.11
COL11A1	J04177	9.86	2.29	
COL1A1	AI743621	2.59	1.79	
ACAN	NM_001135	9.17	2.02	2.65

^*^Negative number indicates decreased expression and positive number indicates increased expression (fold change).

^**^Second microarray using different RNA samples extracted from meniscal explants derived from a different OA patient.

^***^Results of real-time RC-PCR.

**Table 5:** Grades of immunostaining of FCGR3B, LILRB3, FGFR3, and SOX-9.

	Control 12 F	Control 43 M	Control 39 F	OA 77 M	OA 49 F	OA 66 F	OA 70 F	OA 57 M	OA 65 F
FCGR3B	0	1	0	3	3	2	2	1	1
Mean (FCGR3B)	**0.33**			**2.00**					

LILRB3	1	2	1	2	3	3	2	3	3
Mean (LILRB3)	**1.33**			**2.67**					

FGFR3	0	0	0	1	1	1	1	2	1
Mean (FGFR3)	**0.00**			**1.17 **					

SOX-9	3	3	3	2	1	1	1	2	2
Mean (SOX-9)	**3.00 **			**1.50 **					

Ages of the patients are listed in years; M = male; F = female.
